# Indocyanine-Green for Fluorescence-Guided Surgery of Brain Tumors: Evidence, Techniques, and Practical Experience

**DOI:** 10.3389/fsurg.2019.00011

**Published:** 2019-03-12

**Authors:** Steve S. Cho, Ryan Salinas, John Y. K. Lee

**Affiliations:** ^1^Perelman School of Medicine at the University of Pennsylvania, Philadelphia, PA, United States; ^2^Department of Neurosurgery at the Hospital of the University of Pennsylvania, Philadelphia, PA, United States

**Keywords:** indocyanine-green, near-infrared, tumor resection, enhanced-permeability and retention effect, fluorescence-guided surgery

## Abstract

The primary treatment for brain tumors often involves surgical resection for diagnosis, relief of mass effect, and prolonged survival. In neurosurgery, it is of utmost importance to achieve maximal safe resection while minimizing iatrogenic neurologic deficit. Thus, neurosurgeons often rely on extra tools in the operating room, such as neuronavigation, intraoperative magnetic resonance imaging, and/or intraoperative rapid pathology. However, these tools can be expensive, not readily available, time-consuming, and/or inaccurate. Recently, fluorescence-guided surgery has emerged as a cost-effective method to accurately visualize neoplastic areas in real-time to guide resection. Currently, 5-aminolevulinic-acid (5-ALA) remains the only fluorophore that has been approved specifically for fluorescence-guided tumor resection. Its use has demonstrated improved resection rates and prolonged progression-free survival. However, protoporphyrin-IX, the metabolic product of 5-ALA that accumulates in neoplastic cells, fluoresces in the visible-light range, which suffers from limited tissue penetration and significant auto-fluorescence. Near-infrared fluorescence, on the other hand, overcomes these problems with ease. Since 2012, researchers at our institution have developed a novel technique using indocyanine-green, which is a well-known near-infrared fluorophore used traditionally for angiography. This Second-Window-ICG (SWIG) technique takes advantage of the increased endothelial permeability in peritumoral tissue, which allows indocyanine-green to accumulate in these areas for intraoperative visualization of the tumor. SWIG has demonstrated utility in gliomas, meningiomas, metastases, pituitary adenomas, chordomas, and craniopharyngiomas. The main benefits of SWIG stem from its highly sensitive detection of neoplastic tissue in a wide variety of intracranial pathologies in real-time, which can help neurosurgeons both during surgical resections and in stereotactic biopsies. In this review of this novel technique, we summarize the development and mechanism of action of SWIG, provide evidence for its benefits, and discuss its limitations. Finally, for those interested in near-infrared fluorescence-guided surgery, we provide suggestions for maximizing the benefits while minimizing the limitations of SWIG based on our own experience thus far.

## Introduction

Surgical resection of brain tumors remains an important part of cancer care for pathologic diagnosis, relief of mass effect, and survival benefit (1–9). However, radical resections, such as those practiced in other surgical fields, in the brain could result in neurologic morbidity that may outweigh the benefits of surgery (10). Neurosurgeons must thus balance the goals of maximal resection with minimizing neurologic deficits, which is a difficult task. Indeed, gross-total-resection (GTR) rates for intracranial tumors range from <30% for glioblastoma-multiforme (GBM) and other high-grade gliomas (HGG) to ~70% for benign meningiomas or pituitary adenomas, and tumors recur even after perceived GTR (11–16). Therefore, it is of paramount importance that neurosurgeons intraoperatively distinguish neoplasm from benign brain parenchyma and surrounding tissue. In addition to enhanced illumination, magnification, and experienced interpretation of tissue color and texture, neurosurgeons often rely on extra tools in the operating room, such as neuronavigation, intraoperative magnetic resonance imaging (MRI), intraoperative rapid pathology, and/or intraoperative ultrasound (17, 18). However, these modalities can have significant drawbacks, such as brain-shifts for neuronavigation and low availability, high cost, and high false-positive rate for intraoperative MRI (17–21). Recently, fluorescence-guided surgery (FGS) has emerged as a rapid and cost-effective alternative to these techniques.

**Figure 1:**
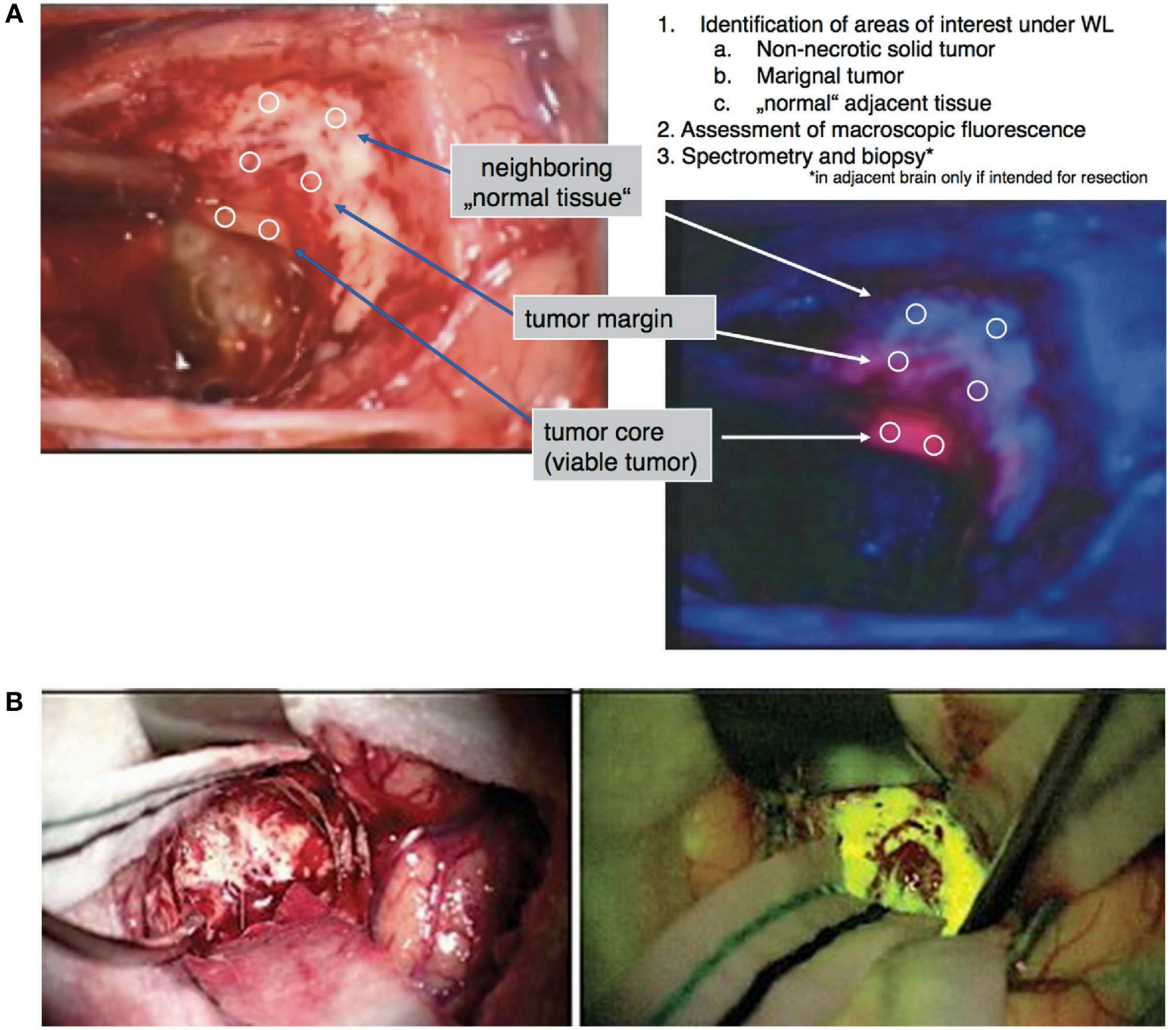
Examples of visible light fluorophores used in brain tumor resection. **(A)** 5-ALA is an oral prodrug that is converted to proptoporphyrin-IX, a visible light fluorophore with red emission, in high-grade neoplastic cells. Under blue-light excitation (400–410 nm), pink areas correspond to areas of neoplasm against the dark-blue background (28). **(B)** Fluorescein is a visible-light fluorophore with yellow emission that highlights areas of blood-brain-barrier damage. When injected intravenously 1–3 h prior to tumor exposure, areas of tumor can be visualized in yellow using yellow-green (460–500 nm) excitation (38).

In June 2017, the US Food and Drug Administration (FDA) approved 5-aminolevulinic-acid (5-ALA) as an agent for fluorescence-guided neurosurgery. First tried in neurosurgery in 1998, 5-ALA is a prodrug that leads to selective accumulation of a fluorophore, protoporphyrin IX (PpIX), in malignant cells, which can then be visualized intraoperatively using blue-light excitation (22–28). In a pivotal randomized-control study, Stummer et al. demonstrated that 5-ALA fluorescence-guided surgery led to a 65% GTR rate, compared to 36% in the control group in patients with high-grade gliomas (29). Other studies have replicated the benefits of 5-ALA in high-grade gliomas, as well as potential benefit in other intracranial tumors, such as meningiomas and pituitary adenomas (30–32). Despite the rigorous evidence that 5-ALA use is advantageous, it has visible-light emission that is significantly absorbed by endogenous fluorophores (i.e., heme), limiting its tissue penetration. Furthermore, the brain has numerous endogenous fluorophores, such as lipofuscin or flavin, with excitation/emission spectra that can overlap significantly with PpIX, reducing contrast between the tumor and the background brain (Figure 1A).

Fluorescein is another FDA-approved fluorophore that has traditionally been used for angiography in ophthalmology but with recent applications in tumor surgery. Studies have demonstrated that a bolus injection of fluorescein during the operation leads to fluorescein accumulating in areas of blood-brain-barrier breakdown in the peritumoral tissue. Fluorescein has demonstrated a range of sensitivity and specificity for intracranial tumors in prior studies, with generally high sensitivity but lower specificity compared to 5-ALA (33–39). Like 5-ALA, fluorescein is a visible-light fluorophore and suffers from limited visualization through tissue and weak contrast from surrounding normal brain (Figure 1B). Some have attempted dual-injections of 5-ALA and fluorescein to enhance detection of neoplastic tissue by increasing the contrast between the neoplastic tissue that uptake 5-ALA and the peritumoral area that uptake fluorescein (40).

More recently, indocyanine-green (ICG), a near-infrared (NIR) fluorophore (peak excitation = 805 nm, peak emission = 835 nm), has demonstrated utility in labeling tumor tissue. Unlike the conventional use of ICG as an angiographic agent, a novel technique using ICG, termed the Second Window ICG (SWIG), has been demonstrated in recent years. Due to the limitations of 5-ALA and fluorescein mentioned earlier, we have focused on NIR fluorophores. Thus, we investigated SWIG in various intracranial tumors, including high-grade gliomas, meningiomas, brain metastases, and pituitary adenomas. In this article, we review the brief history and hypothesized mechanism of action behind SWIG, examine the evidences supporting its use neurosurgery, detail specific operative techniques to minimize errors, and describe our group's practical experience with this novel technique.

## History and Mechanism of Action of SWIG in Intracranial Tumors

Neurosurgeons are familiar with ICG and its role as an angiographic agent since the 1960s. ICG is a small, amphiphilic molecule (<800 daltons) that, when injected intravenously, normally remains within the blood vessel mostly bound to albumin and other plasma proteins. It is removed by biliary excretion and has a very short half-life of <180 s. Due to this short half-life, ICG is usually given as a bolus dose of <0.5 mg/kg and NIR imaging is performed immediately afterwards to delineate the vasculature.

In 1993, Hansen et al. described a technique using ICG boluses to create contrast between neoplastic tissue and brain parenchyma in rat models, rather than just visualizing vasculature (41); this was soon followed by another study by Haglund et al. in rats (42) and in human patients (43). In this last study, patients received a bolus of ICG at 1 mg/kg and then were imaged for up to 10 min after the bolus dosing, allowing ICG to accumulate in the tumor while washing out of the surrounding parenchyma. In patients with HGG, significant contrast between tumor and background was detected, while in patients with low-grade gliomas (LGG), this contrast decreased significantly between 4 and 10 min after ICG injection.

**Figure 2:**
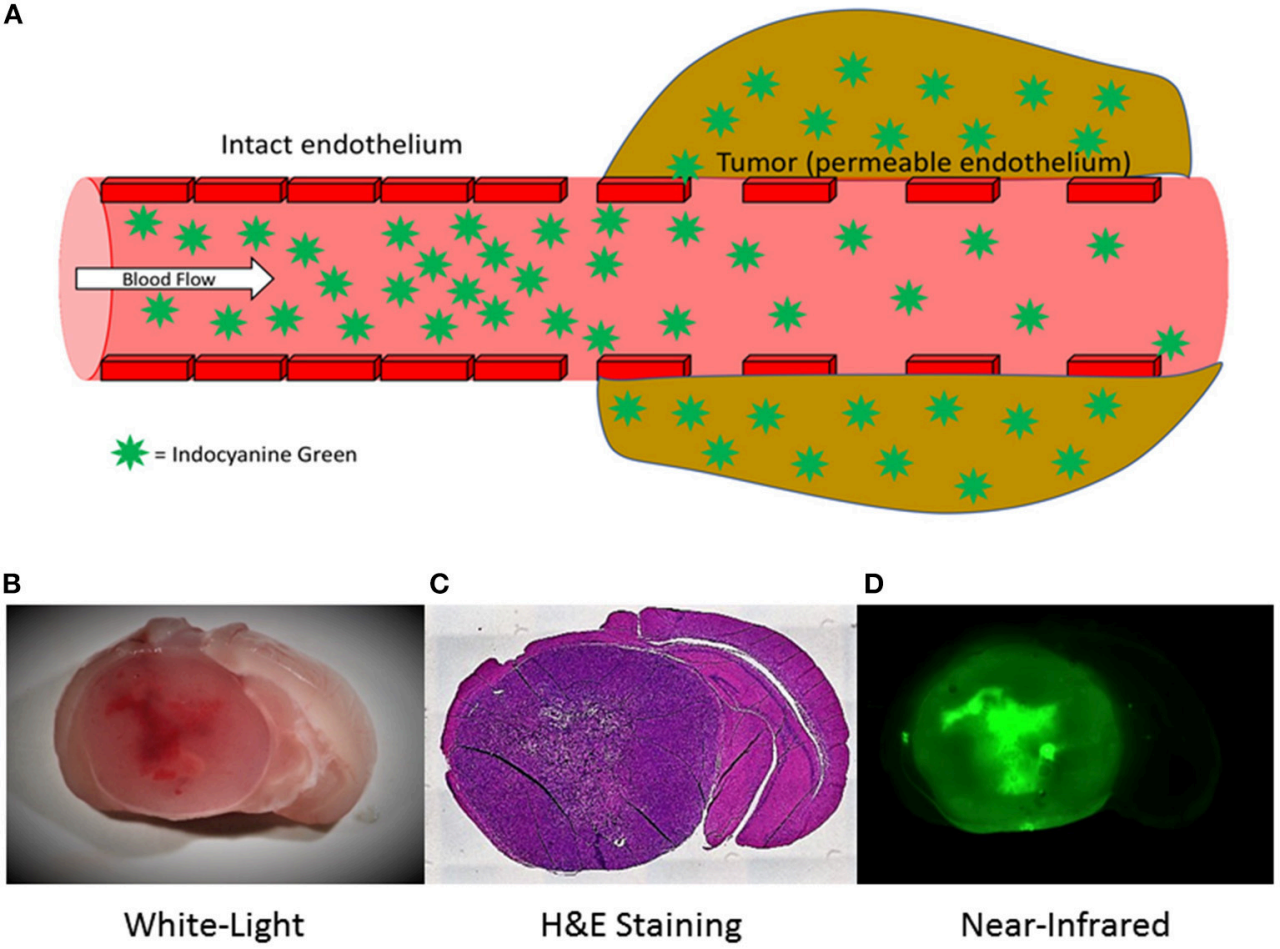
Mechanism of action for Second Window Indocyanine Green (SWIG) **(A)** ICG is hypothesized to accumulate in neoplastic areas via the enhanced permeability and retention effect. In areas of normal brain, with intact endothelium, the ICG remains intravascular and washes away quickly. In areas of tumor, which often have permeable/damaged endothelium, the ICG permeates into the peritumoral tissue and remains in the tissue for a prolonged time (54). **(B–D)** SWIG in murine model of GBM (U87 cells). Under white-light alone **(B)**, it is difficult to visualize the full extent of the tumor, which is demonstrated by hematoxylin and eosin (H&E) staining **(C)**. With SWIG **(D)**, however, both the core of the tumor and the margin are delineated under near-infrared fluorescence.

Finally, in a 2012 study, Madajewski et al. proposed that a high-dose infusion of ICG (7.5 mg/kg) given 24 h prior to surgery allows ICG to accumulate in areas of neoplasm, facilitating detection of residual neoplasm after standard resection in murine flank tumor models (44). This effect was confirmed in a follow-up dose-confirmation study in a murine flank tumor model (45) and was further demonstrated in a murine intracranial tumor model by our lab (46). It has been hypothesized that the accumulation of ICG occurs through the well-described enhanced permeability and retention (EPR) effect, which stipulates that solid tumors possess enhanced vascular permeability due to defective vascular structures, impaired lymphatic drainage systems, and increased permeability mediators (47). ICG then accumulates in these areas of enhanced vascular permeability and can be visualized 24-h later (Figure 2) (48).

## Evidence and Potential Benefits of SWIG

SWIG has been investigated in various intracranial applications (Table 1). We summarize the results below and discuss potential implications.

**Table 1:** Summary of studies investigating near-infrared fluorescence guided neurosurgery with second-window ICG.

**References**	**Tumor type (n)**	**Sensitivity (WL, NIR)**	**Specificity (WL, NIR)**	**PPV (WL, NIR)**	**NPV (WL, NIR)**
Lee et al. (49)	Gliomas (15)	84% 98%	80% 45%	92% 82%	67% 90%
Lee et al. (50)	Metastases (13)	82% 96%	91% 27%	96% 77%	67% 75%
Lee et al. (51)	Meningiomas (18)	82% 96%	100% 39%	100% 71%	78% 88%
Cho et al. (52)	Varied (6)	NA	NA	NA	NA
Jeon et al. (53)	Skull-base Tumors (15)	NA	NA	NA	NA
Cho et al. (54)	Pituitary Adenomas (16)	88% 100%	90% 29%	96% 82%	73% 100%

*NA, Not applicable; NIR, Near-infrared; NPV, Negative-predictive value; PPV, Positive-predictive value; WL, White-light*.

### Intra-Axial Brain Tumors: Gliomas and Metastases

**Figure 3:**
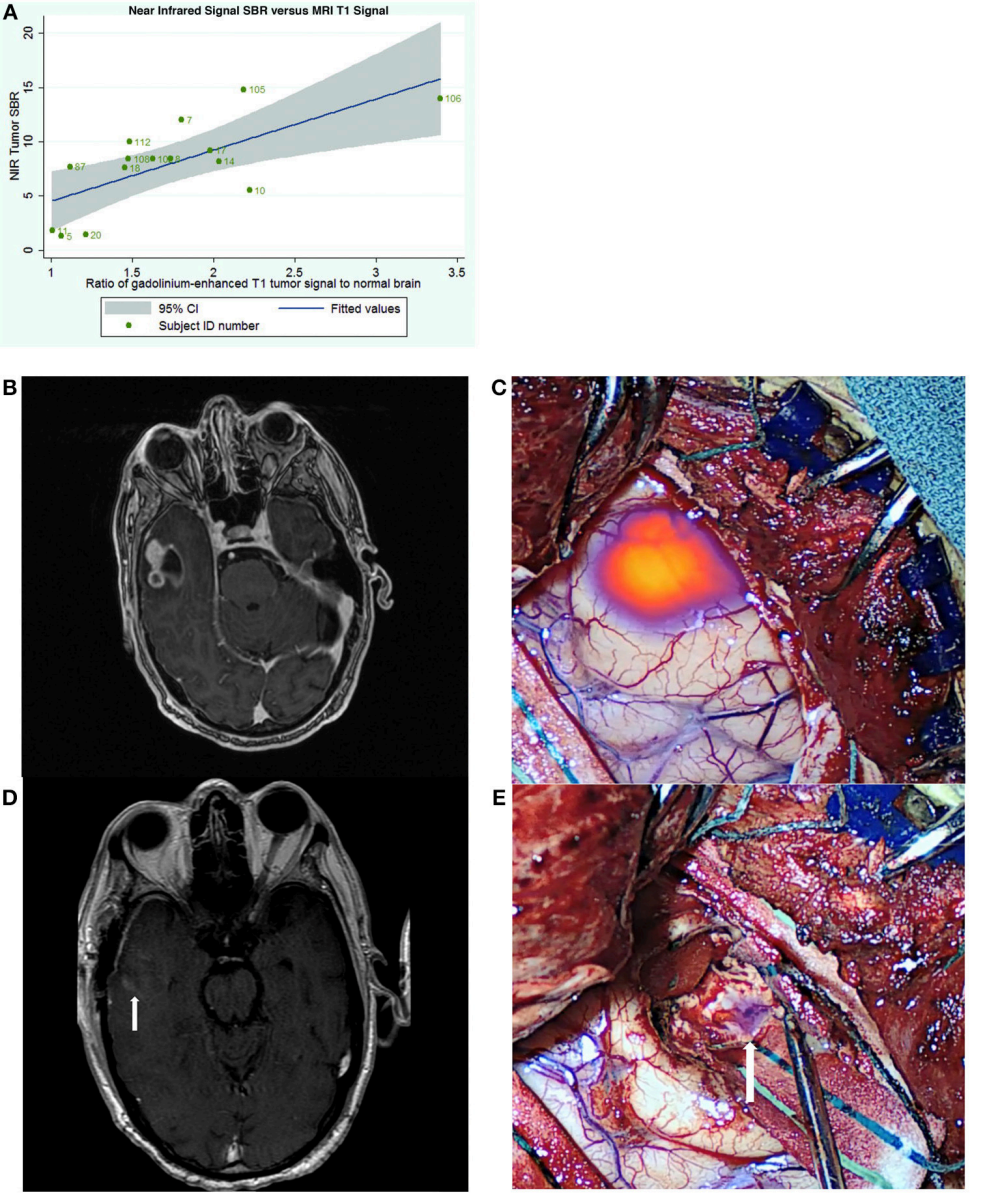
Utility of SWIG in patients with GBM. **(A)** The amount of near-infrared fluorescence detected in tumors after SWIG administration positively correlates with contrast-enhancement on preoperative MRI (*p*-value 0.03), suggesting that SWIG can label contrast-enhancing tissue in real-time (49). **(B,C)** A contrast-enhancing GBM demonstrates strong NIR fluorescence in the operating room after durotomy. This technique does not suffer from brain-shifts, unlike neuronavigation. **(D,E)** After standard resection, NIR imaging of the surgical bed demonstrates no residual areas of strong NIR fluorescence. Postoperative day-1 MRI demonstrates postoperative changes with gross-total resection. Arrows indicate the orientation of the surgical bed. In patients whom post-resection NIR imaging does not demonstrate residual NIR fluorescence, the neurosurgeon can be more confident that gross-total resection has been achieved.

In 2016, we published the results of the first SWIG study in 15 patients with gliomas (49). An important discovery was that the strongest predictor of positive intraoperative NIR fluorescence was contrast-enhancement on preoperative MRI (*p*-value = 0.03) (Figure 3A). NIR fluorescence could be detected from tumors >1 cm deep and in 8 patients, the tumor could be detected prior to durotomy. In contrast-enhancing gliomas (12/15 in this study), SWIG demonstrated 98% sensitivity, 45% specificity, 82% positive-predictive-value (PPV) and 90% negative-predictive-value (NPV) for detecting areas of neoplasm in biopsy specimens, with an area under the receiver operating characteristic curve (AUROC) of 0.715. In comparison, white-light alone was 84% sensitive and 80% specific for neoplasm, with a PPV of 92%, NPV of 67%, and AUROC of 0.822. Updated data from our group that is yet unpublished shows that SWIG has a PPV of 88% and NPV of 63% for margin specimens in contrast-enhancing gliomas, compared to white-light alone with a PPV of 100% and NPV of 38%. Furthermore, data currently under review for publication suggests that NIR imaging of the surgical bed after resection can increase the surgeon's confidence that GTR has been achieved if there are no residual areas of NIR fluorescence (Figures 3B–E).

**Figure 4:**
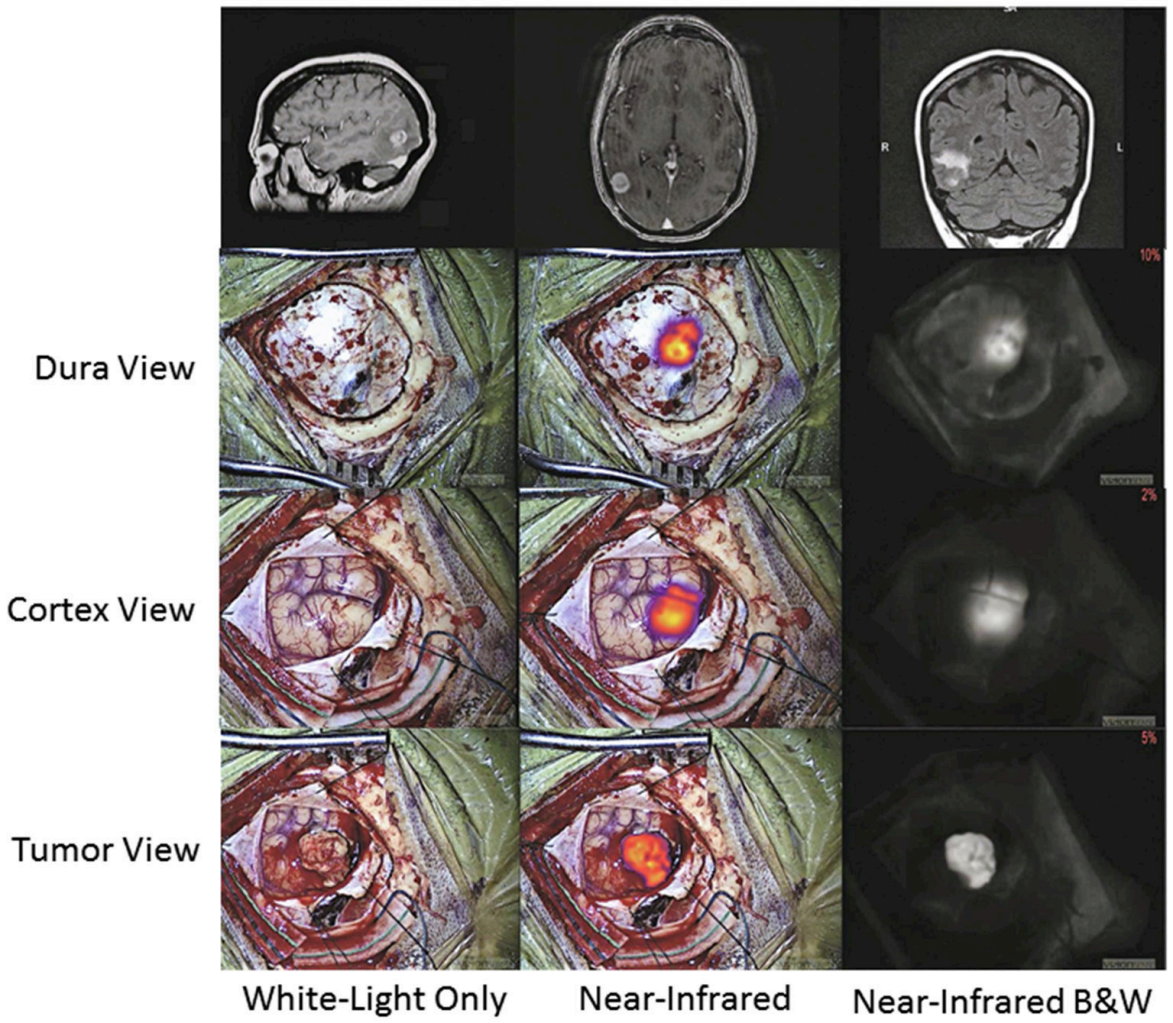
Further clinical applications of SWIG in neurosurgery—trans-dural visualization. In this patient with a subcortical brain metastasis, NIR imaging localized the tumor through intact dura as well as intact cortex. Unlike visible-light fluorescence, NIR fluorescence can travel through tissue up to >1 cm in the brain. Since NIR imaging provides real-time localization of tumor, this can help neurosurgeons better plan the access through the dura and minimize cortical disruption (50).

The utility of SWIG was also investigated in 13 patients with various intracranial metastases in a 2017 study (50). SWIG demonstrated higher sensitivity (96 vs. 82%) and NPV (75 vs. 67%) but lower specificity (27 vs. 91%) and PPV (77 vs. 96%). AUROC with SWIG was 0.619, whereas AUROC with white-light alone was 0.865. NIR signal was visualized through the dura in 11 patients and in patients with sub-surface tumors, NIR signal could be visualized at least to a depth of 6.8 mm below the cortex (Figure 4).

**Figure 5:**
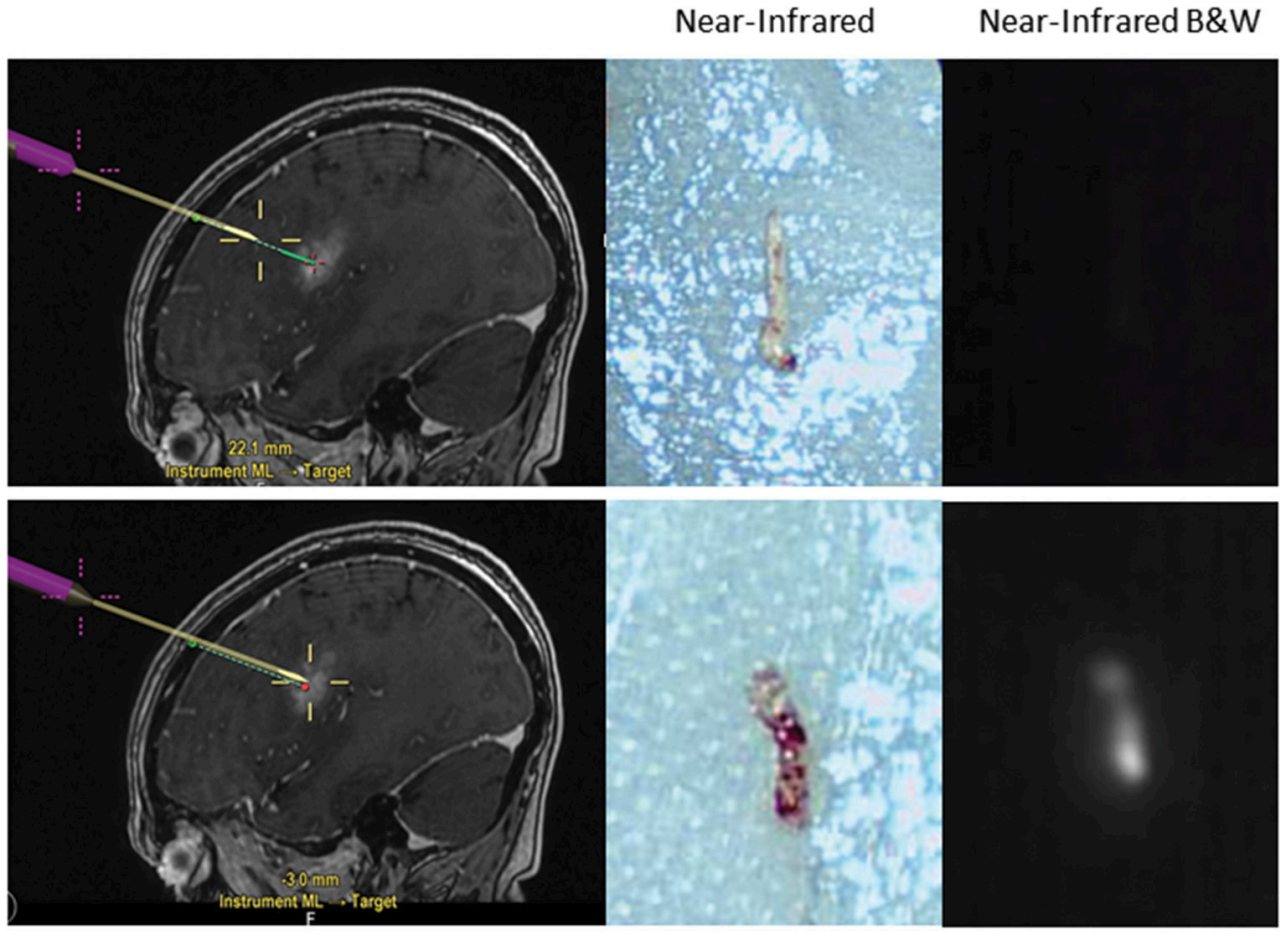
Application of SWIG for stereotactic biopsies. Stereotactic biopsies with SWIG offer rapid confirmation that abnormal tissue was biopsied. When a sample was biopsied outside the contrast-enhancing legion using neuronavigation (top row), it did not demonstrate NIR fluorescence and was normal tissue on the frozen pathology. On the contrary, when a sample was biopsied within the contrast-enhancement (bottom row), it demonstrated NIR fluorescence and pathology diagnosis was GBM. Thus, SWIG offers an affordable, rapid, and accurate adjunct to stereotactic biopsies to reduce operating length.

The latest application for SWIG under investigation is in stereotactic biopsies of intracranial tumors. Currently, stereotactic brain biopsies may have prolonged operative time due to the need for confirmation of abnormal tissue from frozen pathology. If the result is inconclusive, this may require further biopsy specimens in the operating room. However, using SWIG, the neurosurgeon can examine biopsied specimens *ex-vivo* for NIR fluorescence in the operating room. If the tissue is non-fluorescent, chances are very high that the specimen is not neoplastic or otherwise abnormal due to the highly sensitive nature of SWIG for neoplasm, and thus, another biopsy should be taken; conversely, with fluorescent tissue, the surgeon can be confident that abnormal tissue was biopsied (data under review) (Figure 5). Thus, SWIG offers an affordable, rapid, and accurate adjunct to stereotactic biopsies to increase the neurosurgeons' confidence and reduce operating length.

### Extra-Axial Brain Tumors: Meningiomas, Pituitary Adenomas, and Others

SWIG has demonstrated sensitive detection of neoplasm in extra-axial tumors as well. In a 2017 study of SWIG in 18 meningioma patients (15 grade I, 3 grade II), NIR imaging detected strong fluorescence in 14 tumors (51). It was noted that 4 tumors demonstrated “inverse” NIR fluorescence, in which the background signal was higher than the signal within the tumor, and linear regression suggested that time from ICG injection was negatively correlated with NIR fluorescence contrast (*p*-value = 0.022, *R*^2^ = 0.2876); we have hypothesized that this inverse fluorescence may be due to either quenching of ICG that can occur at concentrations >125 ug/L (52) or the slow permeation of ICG out of the neoplastic tissue into the surrounding area after 24 h, although neither has been definitively demonstrated. Overall, SWIG demonstrated 96% sensitivity, 39% specificity, 71% PPV, and 88% NPV for detecting neoplasm in these meningioma patients with an AUROC of 0.677, compared to white-light alone, which showed 82% sensitivity, 100% specificity, 100% PPV, and 78% NPV with an AUROC of 0.911.

**Figure 6:**
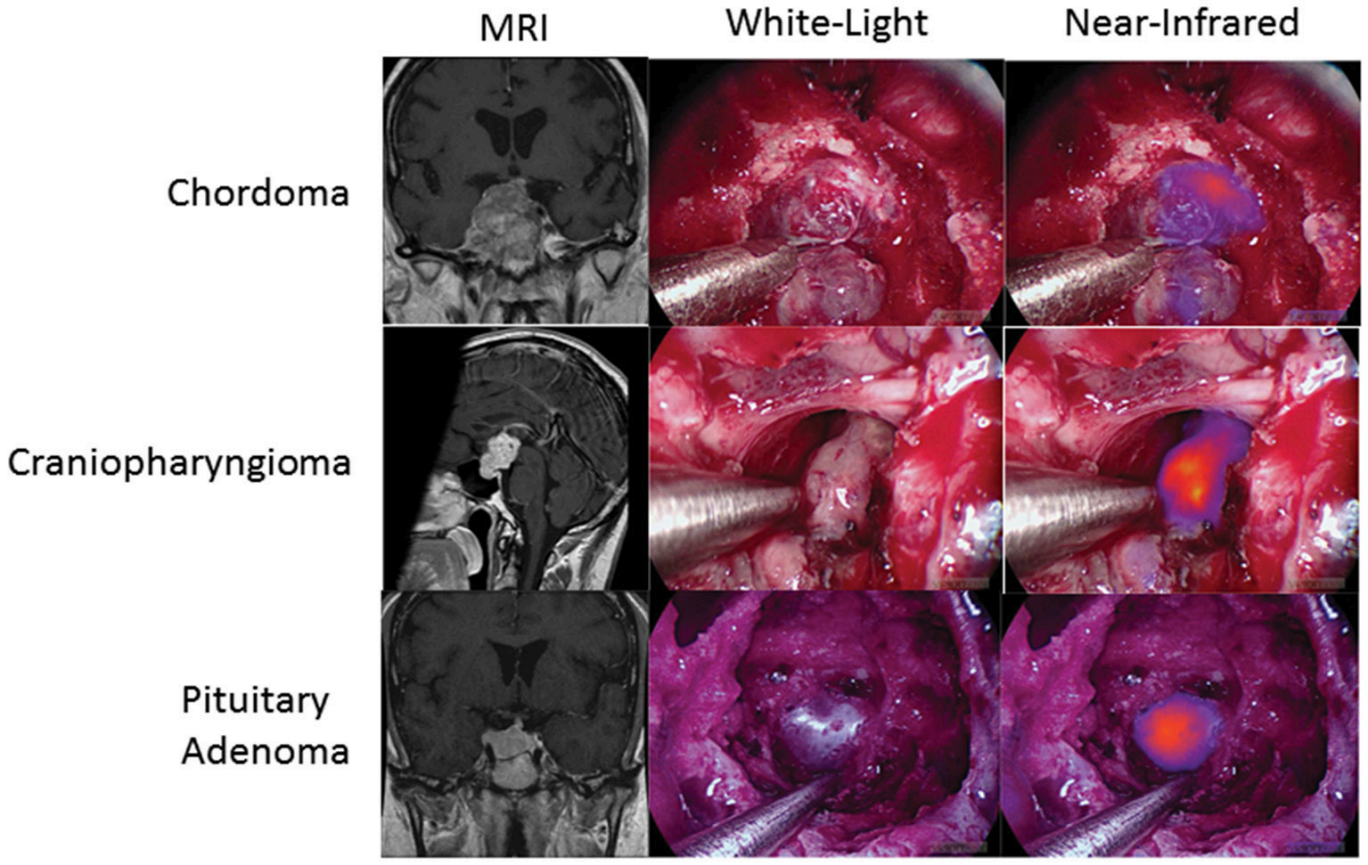
SWIG in extra-axial brain tumors. Patients with chordomas (top row), craniopharyngiomas (middle row), and pituitary adenomas (bottom row) all demonstrate NIR fluorescence with SWIG. Visualization was performed with a NIR-sensitive endoscope system (53).

Finally, SWIG is applicable to skull base tumors, such as pituitary adenomas, chordomas, and craniopharyngiomas, which were accessed via the transnasal/transsphenoidal approach. A 2018 study of 8 pituitary adenomas, 3 craniopharyngiomas, and 4 chordomas demonstrated that all the tumors demonstrated NIR fluorescence (Figure 6) (53). Again, contrast-enhancement on preoperative MRI was the best predictor of NIR fluorescence contrast to normal background (*p*-value = 0.0003). In pituitary adenomas, SWIG demonstrated 100% sensitivity, 20% specificity, 71% PPV, and 100% NPV for neoplasm in biopsy specimens, compared to white-light alone with 90% sensitivity, 100% specificity, 100% PPV, and 83% NPV (*n* = 15). A more recent study shows that SWIG had 100% sensitivity, 29% specificity, 82% PPV, and 100% NPV (*n* = 30), whereas white-light had 88% sensitivity, 90% specificity, 96% PPV, and 73% NPV (*n* = 78) (54).

### Benefits of SWIG in Neurosurgery

Overall, these studies suggest that the main benefit of intraoperative NIR imaging with SWIG is the higher sensitivity and NPV for detecting neoplastic tissue, which may allow surgeons to detect more residual neoplasm at the margins after resection than with white-light alone, increasing the chances of achieving GTR. SWIG studies thus far are all limited by the fact that the scope of surgery was not changed depending on NIR imaging, as SWIG was a technique under investigation at the time. In other words, even if the senior surgeon observed residual areas of fluorescent tissue after standard resection, further resection was not performed. Therefore, it is currently difficult to quantify the effects NIR imaging with SWIG on surgical outcomes. However, given that 5-ALA, with lower sensitivity (~70–90%), similar PPV (>95%), and much lower NPV (19–24%) has demonstrated benefit in increasing GTR rates and progression-free survival (29), it is not unreasonable to expect that fluorescence-guided surgery with SWIG would lead to equally improved, if not better, surgical outcomes compared to surgery with 5-ALA. Furthermore, the results demonstrating correlation between absence of fluorescence and GTR in HGG is encouraging as well. A study to investigate changing the extent of surgery according to NIR fluorescence with SWIG is currently under way and will better elucidate the effects of fluorescence-guided surgery with SWIG on resection rates and surgical outcomes.

In contrast to PpIX or fluorescein, SWIG imaging utilizes NIR fluorescence, leading to great tissue penetration. The longer NIR wavelength allows the photons to travel further in the body, especially in less dense tissue, because there is less absorption by hemoglobin and other proteins (55, 56). Thus, as demonstrated in the above studies, NIR fluorescence from subcortical tumors can be imaged through intact dura and cortex, which is not possible with white-light visualization or with 5-ALA, except under very limited circumstances with specialized equipment (57). Furthermore, since NIR imaging is performed in real-time, SWIG does not suffer from brain shifts and other inaccuracies in directing the surgeon toward the tumor bulk as can be seen in neuronavigation. Both the deeper tissue signal and the real-time feedback can benefit the surgeon in accurately planning access through the dura and minimizing cortical disruption.

Finally, a defining feature of SWIG is its widespread applicability. Unlike receptor-targeted dyes or metabolic dyes, such as 5-ALA, SWIG has demonstrated nearly equivalent utility in a wide variety of intracranial tumors. This, combined with the high availability of ICG, makes SWIG an easily accessible adjunct to the armamentarium of neurosurgeons world-wide.

## Technical Considerations

SWIG is fundamentally different from traditional angiography with ICG or the minimally delayed imaging performed by Haglund et al. In order to take full advantage of the EPR effect, ICG must be administered between 16 and 30 h prior to the surgery (46). Generally, patients receive an outpatient infusion the day prior to surgery. ICG is administered intravenously over 1 h, at a dose of 5 mg per kg of body weight. The patient is closely monitored throughout the duration of the infusion and for 30 min afterwards for any adverse events. Thus, far, no major complications from ICG administration has been reported. The patient then returns the next day for the surgery. Of note, in a few recent cases, including a spinal cord meningioma and peripheral nerve schwannoma, ICG was administered 6–8 h prior to tumor exposure. While NIR imaging detected pathologic tissue in these cases, there is still insufficient evidence to validate this shorter time window in humans.

**Figure 7:**
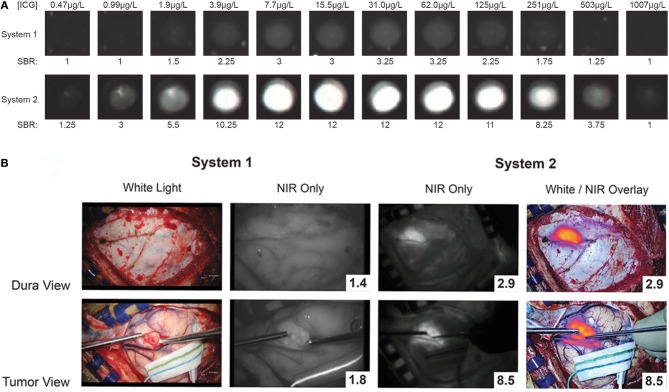
A prior study compared a conventional state-of-the-art microscope (System 1) with a NIR-detecting module to a dedicated NIR-imaging system (System 2). In both *ex-vivo* **(A)** and *in-vivo* visualizations of ICG (**B**; NIR signal-to-background ratio in bottom right corner), System 2 was much more sensitive to NIR fluorescence, with a greater dynamic range. Furthermore, only System 2 was capable of imaging NIR fluorescence through the dura. Thus, for successful surgeries with SWIG, a sensitive, dedicated NIR imaging system is necessary (52).

Intraoperatively, the surgery setup and procedure proceeds as per standard of care, with the one exception of having a dedicated NIR imaging device present in the operating room. While conventional surgical microscopes have add-on modules that allow NIR imaging of ICG, these were designed for ICG angiography with a much higher concentration of ICG. These modules are not sensitive enough to low concentrations of ICG and do not have a wide dynamic range. Our group uses a dedicated NIR exoscope/endoscope system (VisionSense Iridium™), which uses a dedicated excitation laser and an integrated post-acquisition image processing. This allows simultaneous visualization of visual light, NIR signal alone, and an overlaid augmented-reality type display of both signals simultaneously. This system was identified as having superior sensitivity and dynamic range compared to other commercial NIR imaging platforms in a comparison study by DSouza et al. (58). Our group further compared the dedicated NIR exoscope system to a commercial neurosurgical microscope module and demonstrated that for SWIG fluorescence-guided surgery, the dedicated NIR system is more suitable, as it can more reliably detect NIR fluorescence through the dura and detect NIR signal from neoplastic tissue with a higher tumor-to-brain contrast over a larger dynamic range of NIR fluorescent signal (Figure 7) (52). Other dedicated NIR imaging platforms with strong excitation and high-quality image-processing may also be viable options. More sophisticated add-on modules for surgical microscopes are emerging as well.

In order to minimize background and maximize signal detection, ambient light should be minimized. During the SWIG visualization of the procedure, both room and overhead operating lights are turned off as even these lights can lead to some degree of NIR signal detection. Furthermore, careful consideration should be made if simultaneous neuronavigation is utilized. It has been noted that neuronavigation systems utilizing NIR technology for fiducial detection interfere with NIR signal detection. In some systems, a pulsing NIR light is generated from the neuronavigation “camera” and is visualized by the NIR imaging system as a pulsing, high background signal. Therefore, the neuronavigation camera should be directed away from the field while using any SWIG detection system.

After craniotomy but before durotomy, the NIR imaging modules is introduced into the field to assess whether the tumor can be visualized through the dura. In many cases of superficial tumors, the tumor boundary can be easily delineated through the dura. Durotomy is then performed and repeat NIR imaging is performed to confirm tumor location. In cases of deep tumors, neuronavigation is used to locate and expose the tumor. Once the tumor is exposed, NIR fluorescence of the gross tumor specimen is measured. Then, resection of the tumor proceeds in the standard of care manner, without NIR imaging. After the senior neurosurgeon is satisfied with the resection, NIR imaging is again performed to assess for residual fluorescence. Depending on the protocol, any areas of residual fluorescence can be simply recorded, biopsied, or completely removed. Closure is then performed in a standard manner and the patient undergoes postoperative MRI to assess the extent of resection.

## Practical Experience

During our experience with SWIG over the past 4 years, we have come to better understand the various factors that can help or hinder surgeons utilizing SWIG fluorescence-guided surgery, which we detail below.

One of the limitations of SWIG is the higher false-positive detection of neoplasm compared to surgeon judgment with white-light alone. False-positive NIR signal can come from either abnormal or normal tissue. Abnormal tissue, such as neoplasm, inflammation, and necrosis are all associated with endothelial damage and thus, ICG can accumulate in these tissues via the EPR effect, which is not specific to neoplasm, but rather targets areas of blood-brain-barrier breakdown. Since these areas enhance with gadolinium, SWIG will also highlight these areas. The difficulty in distinguishing neoplasm from inflammation and necrosis was noted in murine models as well as canine and human patients with thoracic cancers (59). We have noted a similar phenomenon in neurosurgery, in which SWIG could not distinguish between these abnormal tissues (unpublished data). This is the main limitation of SWIG, as it is not a tumor-targeting dye, and it will likely be difficult to overcome without developing targeting moieties.

False-positive NIR signal can occur in normal tissues as well, especially in the skin, mucosa, and dura. It is believed that due to the negative charges on ICG, it can bind nonspecifically to these tissue with a low affinity, although this has not yet been definitively demonstrated as the cause. Thus, in many cases, we have observed NIR signal from skin, mucosa, and/or dura that are certainly not neoplastic. There are two steps that the surgeon can take in the operating room to minimize these false-positive signals.

**Figure 8:**
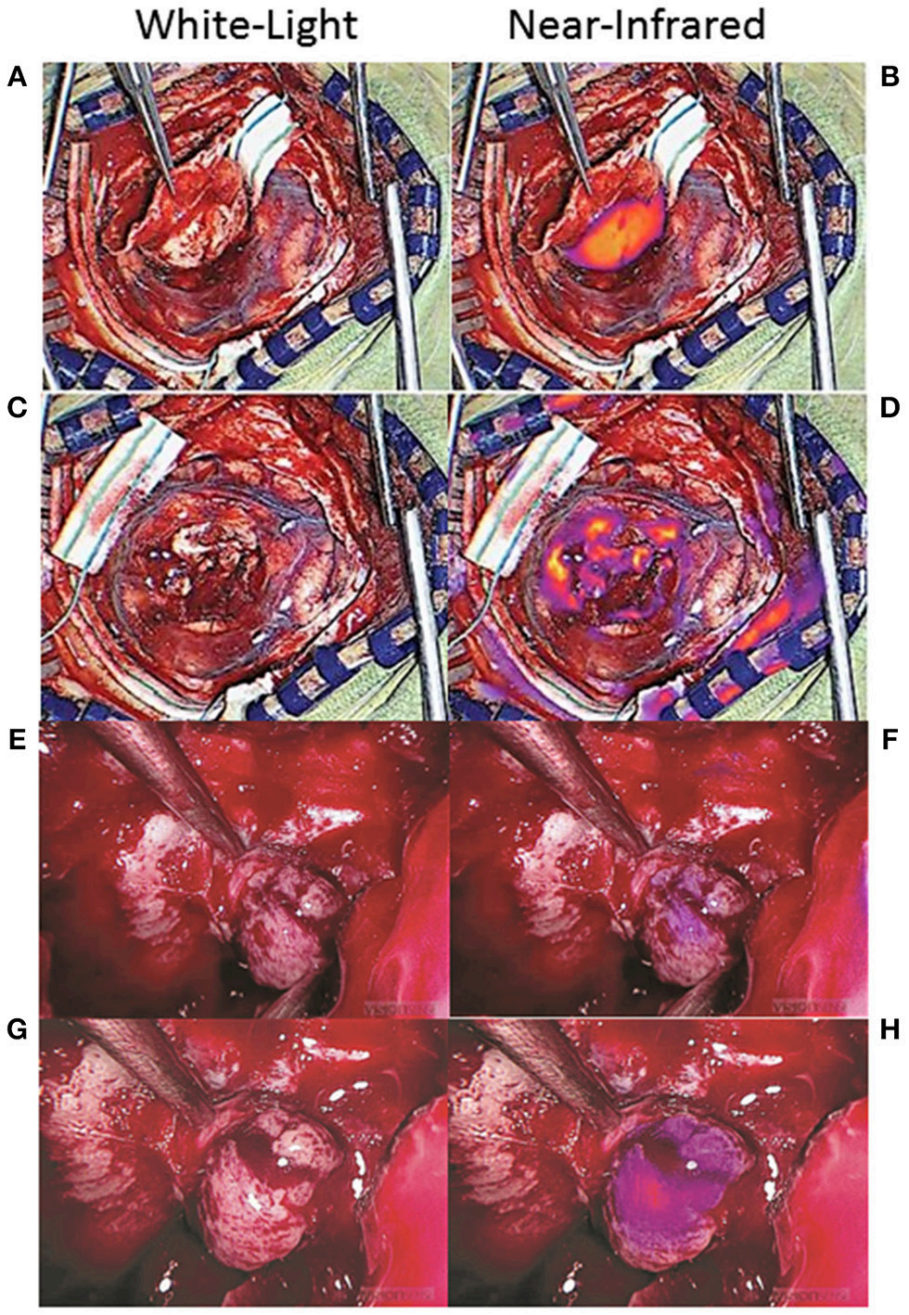
False-positive NIR signal using SWIG. There are two main sources of false-positive signals that may arise during NIR imaging: high gain settings and small distance between the NIR sensor and the tissue. **(A–D)** Some NIR imaging systems have adjustable gain (NIR sensitivity) that need to be fixed in order to avoid detecting false-positive signals. When the tumor was first exposed in this patient with a meningioma **(A)**, the gain was at 15% and strong NIR fluorescence was detected from the tumor **(B)**. After resection **(C)**, NIR fluorescence was again detected, but the gain was at 71% **(D)**. Once the gain was adjusted down to 15%, those areas of NIR fluorescence disappeared, suggesting that the NIR signal had been false-positives (51). **(E–H)** In endoscopic fluorescence-guided surgery, the distance between the scope and the tissue of interest is important as well. Since optical signal decays as a factor of distance-squared, bringing the scope in close proximity to any tissue can result in fluorescence detection. This patient with a pituitary adenoma received a folate-receptor targeted near-infrared dye, but postoperative immunohistochemistry demonstrated no folate-receptor expression; thus, the tumor should not have demonstrated fluorescence. When the tumor was visualized from a distance **(E,F)**, there was minimal NIR signal; however, when the scope was brought closer to the tissue **(G,H)**, the tumor seemed to fluoresce. Thus, maintaining the proper distance between the endoscope and the tissue is of paramount importance in avoiding false-positive NIR signals (61).

Many NIR imaging systems have variable gains, which reflect the sensitivity of the camera sensor to the emission photons, that can be adjusted and fixed by the operator. The higher the gain, the more sensitive the camera, and the weaker the signals that can be detected. Thus, a gain that is set too high can result in detection of NIR fluorescence that isn't true neoplasm (Figures 8A–D) (51). One way to avoid this problem is to note the gain setting when the tumor is first exposed and then to fix the gain at that level throughout the rest of the surgery. The surgeon can then be confident that any NIR fluorescence that is detected is at the same level of fluorescence as the tumor, increasing the likelihood that the area of fluorescence is neoplastic.

Another factor that can contribute to false-positive NIR signals is the distance between the sensor and the tissue. This is more of a factor with endoscopic surgeries, as the scope is often brought to very close proximity to the tissue of interest for optimal visualization. Since optical signal decays as a factor of distance-squared, simply halving the distance between the endoscope and the tissue of interest can elevate the detected NIR signal 16-fold (4-fold increase in excitation strength, 4-fold increase in emission detection). Thus, similar to the gain above, having the endoscope too proximal to the tissue can cause falsely elevated NIR signal to be detected. In order to avoid this, the surgeon should attempt to visualize areas of suspected NIR fluorescence from a set distance. With the VisionSense Iridium endoscope in transsphenoidal surgeries for pituitary adenomas using a related NIR dye, we demonstrated that having the endoscope at a distance that maintains the distance between the two medial opticocarotid recesses to be <50% of the field of view is ideal for minimizing false-positive NIR signals (Figures 8E–H) (60, 61). Endoscopes that offer more precise distance measurements could further help the neurosurgeon to acquire more accurate NIR signals.

**Figure 9:**
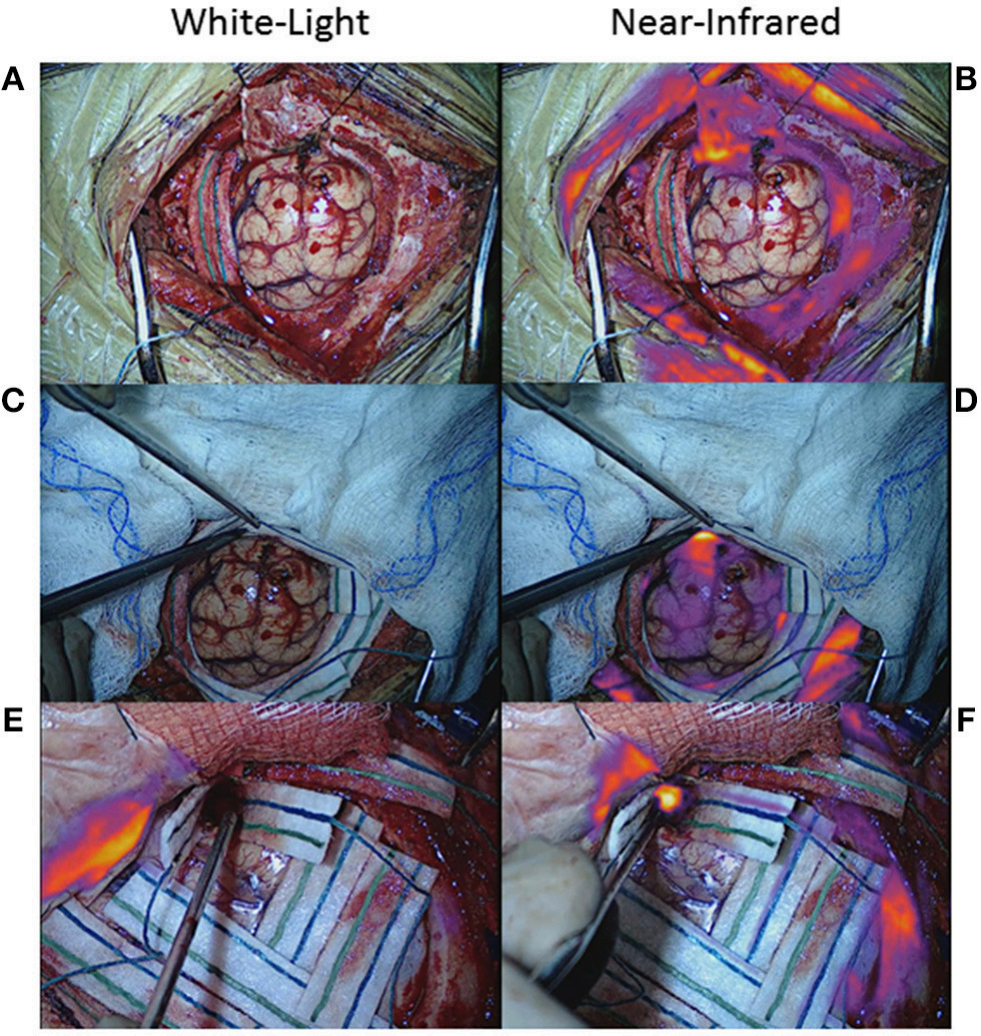
False-negative NIR signal using SWIG. There are two main sources of false-negative signals that may arise during NIR imaging: surrounding, nonspecific NIR signal and blood/fluid. **(A–D)** Upon durotomy in this patient with a GBM, strong but nonspecific NIR signal was observed in the skin and dura, which masked any NIR signal coming from the actual tumor. Once the nonspecific signals were obscured by the surgeon, the NIR fluorescence from the tumor could be detected properly. **(E,F)** Blood and other fluids can obscure NIR fluorescence. In this patient, after standard resection, no NIR fluorescence was detected in the surgical bed initially. However, after removing blood and fluid, the underlying NIR fluorescence was detected.

Furthermore, there are two factors that can hinder the surgeon's ability to detect true-positive NIR fluorescence: strong signal in the surrounding normal tissue and pooled blood. If the gain is properly set as described above, the surgeon should be able to detect residual areas of NIR fluorescence that is at the same level of fluorescence as the tumor, and thus, likely to be residual neoplasm. However, if there is a significant amount of fluorescence in the skin, mucosa, or dura surrounding the surgical bed, the NIR camera may not properly detect smaller areas of fluorescence. If the surrounding area is covered with surgical towels or gauze to isolate the surgical bed, small areas of residual fluorescence can be detected more easily (Figures 9A–D). Pooled blood can also often obscure areas of NIR fluorescence by physically blocking the excitation and emission lights (Figures 9E,F). Thus, when imaging for NIR fluorescence, it is important to achieve hemostasis to clear the field of blood and place the tissue of interest in direct line of the excitation light.

Overall, fluorescence-guided surgery using SWIG has technical intricacies that may require a learning curve. We have detailed the steps that neurosurgeons can take in the operating room to both minimize false-positive signals and to elucidate true-positive signals.

Finally, a consideration for neurosurgeons interested in the technique is the cost of SWIG. As mentioned previously, SWIG requires sensitive NIR imaging capabilities, which is best achieved with a dedicated imaging system, such as the VisionSense Iridium; this system currently costs approximately $100–150 K depending on whether the endoscope function is available. The cost of ICG may vary by institution; a typical patient may need 350–400 mg of ICG (5 mg/kg bodyweight).

## Future Research

There are two main directions in which future research can improve NIR fluorescence-guided neurosurgery. One is improving the specificity of NIR imaging. Despite its potential benefits in the operating room, SWIG is limited mainly by its low specificity as it works through the EPR effect, which is not tumor-specific. One way to overcome this limitation may be to conjugate novel NIR dyes that target specific receptors on neoplastic cell surfaces. In fact, multiple such conjugates are currently being investigated in preclinical and clinical trials for different intracranial tumors. For instance, folate-receptor overexpression on nonfunctional pituitary adenomas and meningiomas may provide targets for sensitive and specific detection of these tumors in real-time (54, 60–62). An alternative is to use imaging apparatus with higher resolution for fluorescence, which may better distinguish true areas of fluorescence from areas of false-positive fluorescence. This has been demonstrated in murine models with fluorescein and ICG using confocal microscopy and may become applicable clinically (63, 64).

Another limitation of NIR imaging in the operating room is the low sensitivity of the imaging equipment. It is well-acknowledged, and our group has demonstrated, that conventional surgical microscopes with add-on NIR modules are significantly less sensitive for NIR fluorescence, especially at lower concentrations, than dedicated NIR imaging modules. Integrating the high NIR sensitivity into conventional microscopes will hugely increase the applicability of NIR fluorescence-guided neurosurgery, further increasing the potential benefits of this novel technique to improve patient outcomes.

## Conclusion

Fluorescence-guided surgery with SWIG is a widely applicable technique that allows neurosurgeons to visualize tumors and residual neoplasm at the margins with greater sensitivity than with white-light and microscopy alone. In this review, we have explained the mechanism of SWIG and its potential benefits, as well as steps that neurosurgeons can take to maximize the utility of this novel technique. Results with SWIG thus far are encouraging and suggest potential benefit in improving patient outcomes in neurosurgery.

## Data Availability

The datasets generated for this study are available on request to the corresponding author.

## Author Contributions

SC drafted the manuscript and prepared the figures and tables. RS and JL provided critical revisions for the manuscript. All authors agree to be accountable for the contents of this work.

### Conflict of Interest Statement

JL has stock options in VisionSense, which owns VisionSense Iridium™used extensively in the studies reviewed here. The remaining authors declare that the research was conducted in the absence of any commercial or financial relationships that could be construed as a potential conflict of interest.
